# Effect of Dimensions and Agglomerations of Carbon Nanotubes on Synchronous Enhancement of Mechanical and Damping Properties of Epoxy Nanocomposites

**DOI:** 10.3390/nano8120996

**Published:** 2018-12-01

**Authors:** Tingting Wang, Bo Song, Kun Qiao, Yudong Huang, Li Wang

**Affiliations:** 1 School of Mechanical & Electrical and Information Engineering, Shandong University, Weihai 264209, China; wt19900923@163.com (T.W.); 13953108281@139.com (K.Q.); 2 Marine college, Shandong University, Weihai 264209, China; 3 MIIT Key Laboratory of Critical Materials Technology for New Energy Conversion and Storage, School of Chemistry and Chemical Engineering, Harbin Institute of Technology, Harbin 150000, China; ydhuang.hit1@aliyun.com

**Keywords:** C-MWCNT, dimensions, agglomeration, dispersion, nanocomposite, synchronous enhancement

## Abstract

In order to achieve simultaneous enhancement of mechanical and damping properties, epoxy resin nanocomposites reinforced with a series of carboxylic multi-walled carbon nanotubes (C-MWCNTs) with different dimensions were prepared. A solution-based high-speed shear dispersion method was developed. The dispersion mechanism of carbon nanotubes was studied, and the degree of dispersion difficulty of carbon nanotubes with different dimensions was evaluated by theoretical calculation, and the minimum size of agglomerates for dispersion based on the mechanism of rupture was deduced. Then, the effect of synchronous enhancement on the mechanical and damping properties was tested by experiment. The effects of dimensions and agglomerations on the tensile properties, damping properties, and glass transition temperature (T_g_) of the nanocomposites were investigated. The ranking of dispersion difficulty was verified using the deviations between predicted and experimental tensile modulus. The experimental results showed that the effects of synchronous enhancement on the mechanical properties and damping capacity of two kinds of specimens were remarkable and the only drawback was that their T_g_ showed the maximum decrease. Further studies indicated that C-MWCNTs with large aspect ratios and large specific surface areas possessed better effects on synchronous enhancement, but caused a decrease in the glass transition temperature, while agglomeration had the opposite effect. The results of this work would be helpful for preparing improved structural damping integrated composites.

## 1. Introduction

Due to the widespread use of composite materials in aerospace, automobile, and other fields, their vibration and noise reduction performance is becoming more and more important. Damping is a key parameter in the control of structural vibration and noise [1]. The damping properties of composites are largely derived from the matrix [2]. Therefore, it is feasible to improve the damping properties of composites by modifying the damping of the matrix. For the damping integration of composite structure, the filler should be able to simultaneously improve the mechanical properties and damping properties of the matrix, without affecting the light weight of the composite material.

Carbon nanotubes (CNTs) are ideal damping fillers. CNTs were first discovered by Japanese scholar Iijima under a high-resolution transmission electron microscope in 1991 [3]. Subsequently, it was found that the modulus of carbon nanotubes was about 1 TPa and the strength was about 14 GPa using simulation [4,5], indirect measurement [6], and direct measurement [7]. Carbon nanotubes are characterized by a large aspect ratio, large specific surface area, pore structure, quasi-one-dimensional structure, and nanometer size. The mechanical properties of polymers can be significantly improved by the addition of a small amount of CNTs. Chang et al. [8] prepared single-walled carbon-nanotube-reinforced thermoplastic fibers (polypropylene, PP) and found that the modulus of the fiber was significantly improved from 0.4 to 1.4 GPa. In the work of Xu et al. [9], the mechanical properties of epoxy resin were reinforced by multi-walled carbon nanotubes (MWCNTs) and a significant increase in flexural modulus from 4.2 to 5 GPa was observed with the addition of only 0.1 wt. % of MWCNTs. In terms of damping properties, Xu et al. [10] found that, compared with silicone rubber, carbon nanotubes had excellent viscoelasticity properties over a wide range of temperatures. Moreover, they produced more energy dissipation than silicone rubber under the conditions of periodic strain. In addition, Zhou et al. [11] reported that the stick-slip frictional motion between the nanotubes and the resin could increase energy consumption. Buldum [12] also pointed out that debonding slippage occurred between carbon nanotubes and the matrix under external force, resulting in interface friction, which in turn led to energy dissipation. Nanometer-sized carbon nanotubes (CNTs) could increase the interfacial area dramatically between fillers and the polymer matrix. The pull-out failure similar to that of scabbard was observed in the matrix reinforced by multi-walled carbon nanotubes, which indicated that there was also sliding between the inner and outer walls of multi-walled carbon nanotubes [13]. Therefore, multi-walled carbon nanotubes (MWCNTs) show the potential to synchronously enhance the mechanical properties and damping properties of the polymer matrix.

However, there are three problems that still need to be solved to realize the integrated design of structural damping using MWCNTs. 

Firstly, which dimensions of MWCNTs should we choose to achieve synchronous enhancement of mechanical properties and damping properties? Many researches were carried out to study the effect of MWCNT dimensions on the mechanical properties of nanocomposites. Dubnikova et al. [14] studied the effect of MWCNT dimensions on the morphology, mechanical reinforcement, and electrical properties of PP-based composites. The influence of MWCNT dimensions on the mechanical properties and electrical conductivity was evaluated by Su et al. [15]. Singh et al. [16] investigated the effect of length on the mechanical, electrical, and electromagnetic interference shielding of MWCNT/epoxy nanocomposites with two different lengths of MWCNTs. Nevertheless, there are few studies on the effect of MWCNT dimensions for the synchronous enhancement of mechanical properties and damping properties of nanocomposites. Similarly, the influence of agglomeration on mechanical properties was frequently reported, but studies on the influence of agglomeration on damping properties are relatively scarce.

Secondly, the dispersion is always an urgent problem in the application of carbon nanotubes. A variety of dispersion methods, such as ultrasonic, ball milling, and mechanical agitation were previously applied. The solution processing of composites which mixed the polymer and nanotube in a suitable solvent and cooperated with the above dispersion methods was developed to improve dispersion [17,18,19]. Arun et al. [20] reported an approach by simultaneously applying ultrasonic waves and shear force generated by an axial flow impeller to obtain a superior level of dispersion of multi-walled carbon nanotubes in epoxy. Then, more approaches were developed to improve the dispersion of carbon nanotubes, among which the functionalization of carbon nanotubes was the most effective. Strong acids and other oxidizing agents were used to generate various functional groups such as carboxyl, ketone, etc., and amination, fluorination, etc. were introduced via further reactions. In addition, non-covalent functionalization approaches, such as special polymers [21] and sodium dodecyl sulfate (SDS) [22], were also used to improve the dispersion of MWCNTs. Studies focusing on the dispersion of CNTs using functionalization were frequently reported in recent publications. Gröschel et al. [23] reported that the selective adsorption of Janus micelles (JMs) on MWCNTs changed the compatibility between CNTs and the matrix, which facilitated the dispersion of MWCNTs. Parveen et al. [24] utilized Pluronic F-127 as a novel dispersing agent to achieve a short dispersion route in cementitious composites. In our experiment, a solution-based high-speed shear dispersion method was developed, and functionalized MWCNTs and a surfactant (BYK 9077) were also applied in the dispersion process.

Finally, the enhancement effect is greatly affected by the dispersion state of MWCNTs. Song et al. [17] compared the properties of MWCNT-reinforced epoxy nanocomposites prepared via direct dispersion and solvent dispersion. The results showed that the properties of MWCNT/epoxy nanocomposites with good dispersion (solvent dispersion) were superior to the nanocomposites with poor dispersion (direct dispersion). In our experiment, the difficulty degree of dispersion of MWCNTs with different dimensions is different using certain dispersion processes, and non-uniform dispersion may occur. The influence of non-uniform dispersion must be considered when analyzing the enhancement effect of MWCNT dimensions. Microscopic observation is a common method of determining the uniformity of dispersion. Rahman et al. [25] studied the dispersion state of amino-functionalized MWCNTs in epoxy resin using TEM. Zeiler et al. [26] analyzed the length distribution of MWCNTs using TEM in the study of the withholding effect of fiber on CNTs. In addition, the micromorphology of fracture surfaces obtained with SEM was used to roughly assess the state of CNTs in a large number of studies [27,28]. In this paper, a method combining theoretical analysis and experimental verification was applied to evaluate the dispersion state of carbon nanotubes with different dimensions. The cohesion and van der Waals forces were used to characterize the difficulty of dispersion of MWCNTs with different dimensions. Then, the prediction of the difficulty degree of dispersion was confirmed, and it was verified whether the uniform dispersion of different dimensions was achieved by comparing the experimental and predicted values of tensile modulus.

In summary, in order to synchronously enhance the mechanical properties and damping properties, five kinds of carboxylic MWCNTs (C-MWCNTs) with different dimensions were selected to prepare MWCNT-reinforced epoxy nanocomposites by mixing nanotubes with epoxy resin using high-speed shear dispersion. Considering the significant influence of dispersion on the properties, a method combining theoretical analysis and experimental verification was used to evaluate the dispersion state of carbon nanotubes with different dimensions. The mechanical properties, damping properties, and glass transition temperature of nanocomposites were studied experimentally. The effects of C-MWCNT loading, length, diameter, aspect ratio, and agglomeration on the mechanical properties, damping properties, and glass transition temperature of the nanocomposites were also investigated.

## 2. Methods and Materials

### 2.1. Materials

The five different kinds of C-MWCNTs used in this experiment were purchased from Xianfeng Nanotechnology Co., Ltd., Nanjing, China. Their basic technical parameters are shown in Table 1. All of them were prepared using chemical vapor deposition (CVD) and were obtained as a black powder in a dry state with a density of 2.1 g/cm^3^. An epoxy resin system (HT723) developed by Wells Advanced Materials Co., Ltd. (Shanghai, China) was used as the matrix material. The resin system had two components, HT723A and HT723B (hardener), which were mixed at a weight ratio of 100:30. The basic physical and mechanical properties of the epoxy resin are shown in Table 2.

### 2.2. Preparation of Composites

**Figure 1:**
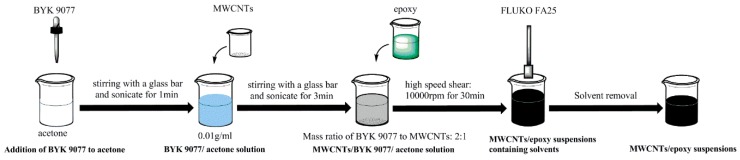
The dispersion process of multi-walled carbon nanotubes (MWCNTs).

In this study, a solution-based high-speed shear dispersion method was developed. The solvent was acetone, and BYK 9077 (BYK, Wesel, Germany) was added as the surfactant. The addition of BYK 9077 enabled the carbon nanotubes to be fully encapsulated by the epoxy resin, reduced the agglomeration of carbon nanotubes, and prevented the re-agglomeration of carbon nanotubes in the dispersion process. The preparation process used in this experiment is shown in Figure 1. Firstly, BYK 9077 was dispersed in the acetone solvent ultrasonically (1 min) with a concentration of 0.01 g/mL. Then, carbon nanotubes were added into the mixture solution of BYK 9077/acetone and the volume of the required solution was calculated according to the mass ratio of BYK 9077 to carbon nanotubes (2:1). The CNT/BYK 9077/acetone solution was also obtained using ultrasonic dispersion (3 min). Finally, the epoxy resin was added to the CNT/BYK 9077/acetone solution for high-speed shear dispersion. A high-speed shear dispersion agitator (FA25, FLUKO, Shanghai, China) was used to stir the mixed solution at a speed of 10,000 rpm for 30 min. The entire device was placed into a chilled water tank during stirring to prevent the temperature from being too high, which would influence the dispersion effect. After the suspension was obtained, most of the solvent was removed by vacuum distillation at 35 °C for 15 min. The remaining suspension was then magnetically stirred in a water bath at 53 °C for 8 h. Finally, the solvent was removed completely in a vacuum oven for 3–5 days. A curing agent was added to the suspension with a mass ratio of 100:30, followed by mechanic stirring for 5 min. The air from the epoxy resin was extracted in a vacuum oven for 20 min and then cast into a steel mold. The curing procedure was 35 °C for 30 min, 50 °C for 120 min, and 120 °C for 180 min. Eight different types of specimens, including control samples, were prepared. Each sample was labeled in the form of “length, diameter, loading” to represent the type and loading of carbon nanotubes in the sample, as shown in Table 3.

### 2.3. Testing and Characterization

Scanning electron microscopy (SEM) of the MWCNT powder was carried out using a Nova NanoSEM 450 (FEI, Hillsboro, OR, USA). The tensile tests of nanocomposites were performed using a Model E45 universal tester (MTS, Shenzhen, China) based on the procedure of American Society for Testing and Materials (ASTM) D638. A dumbbell-shaped specimen was selected for the tensile test, with a total length of 165 mm, widths of 13 mm in the middle and 19 mm at both ends, a gauge length of 50 mm, and a thickness of 3.2 mm. At least five valid data points were obtained from seven samples in each group. The fracture surfaces of tensile specimens were also studied using SEM. The glass transition temperature was measured using a dynamic thermomechanical properties test (DMA Q800, TA, New Castle, PA, USA). The measurement method was ramp temperature frequency scanning; the temperature range was 25–180 °C, and the test frequency was 1 Hz. The sample dimensions were 60 × 10 × 3.2 mm, and the testing mode was three-point bending with a span of 50 mm. Three samples were tested in each group. Damping performance was characterized by loss factor, which was also determined using the DMA test. The test mode and the sample were the same as for the dynamic thermomechanical properties test. The test method was step temperature multi-frequency scanning; the temperature range was 25–35 °C, and the frequency range was 1–50 Hz.

## 3. Results and Discussion

### 3.1. Morphologies of MWCNT Agglomerates

**Figure 2:**
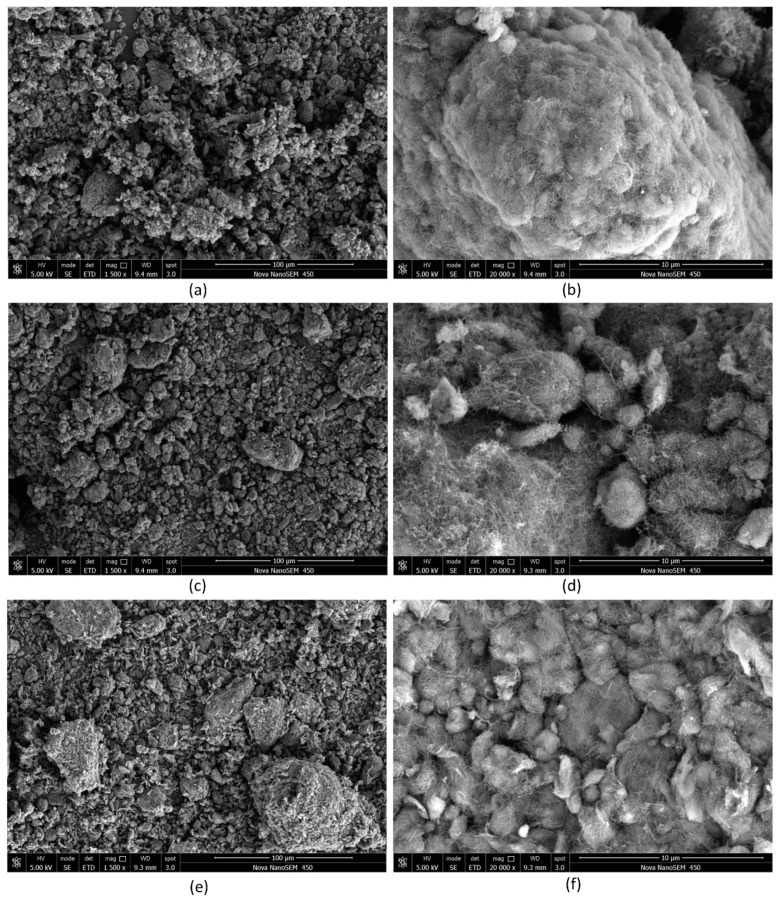
Microscopic morphologies of MWCNT powders with three different lengths (50 μm: low magnification (**a**); high magnification (**b**); 10–30 μm: low magnification (**c**); high magnification (**d**); 0.5–2 μm: low magnification (**e**); high magnification (**f**)).

The microscopic morphologies of carbon nanotubes with three different lengths (50, 10–30, and 0.5–2 μm) are shown in Figure 2. At low magnification, MWCNTs prepared using CVD were all in an agglomerated state, but the morphologies of agglomerates with different lengths were different. Granular agglomerates were predominant in MWCNTs with a length of 50 μm (Figure 2a), but some bundles of agglomerates were also presented and there was obvious adhesion between the agglomerates. MWCNTs with a length of 10–30 μm (Figure 2c) were in the form of granules without bundle agglomerates, and the surface of the particles was relatively smooth. There were larger agglomerates 100 μm in size in MWCNTs with a length of 0.5–2 μm (Figure 2e). Granular agglomerates also accounted for the majority, but some of the particles were slender. At high magnification, it was found that the larger agglomerates in the three kinds of MWCNTs were composed of smaller agglomerates. As shown in Figure 2b,d, the surface of small agglomerates in MWCNTs with a length of 50 μm was rough, and the adhesion between small aggregates was tight. On the other hand, the surface of MWCNTs with a length of 10–30 μm was smoother, and the bonding between them was relatively loose. In Figure 2f, some of the small agglomerates of MWCNTs with a length of 0.5–2 μm were spindle-shaped, which was different from the shape of agglomerates in the first two kinds of MWCNTs.

### 3.2. Dispersion of MWCNTs

The state of carbon nanotubes in the cured matrix greatly affects the properties of the nanocomposites. However, there are relatively few theoretical and experimental studies focused on the dispersion process. The length distributions of carbon nanotubes (CNTs) after several different dispersion processes were analyzed and compared by Graf et al. [29]. Kasaliwal et al. [30] investigated and recorded the areas of agglomerates at different dispersion times. The dispersion mechanism of CNTs was studied in detail by the changing area of agglomerates. 

There are two main dispersion mechanisms of agglomerates: rupture and erosion [30,31,32]. Classically, rupture and erosion occur at the same time, but one dispersion mechanism generally dominates the process under a certain condition. In the rupture mechanism, the crack propagates through the agglomerates, which causes large agglomerates to disperse rapidly into smaller agglomerates, and greatly reduces the size of the agglomerates. In the erosion mechanism, the free carbon nanotubes or small flaky carbon nanotubes peel off from the larger agglomerates due to infiltration and permeation of the solution. These two mechanisms have their own advantages and disadvantages. The rupture mechanism is fast, but causes breakage of the dry agglomerate core. This in turn leads to the undesired breakage of tubes, reducing their aspect ratio. The erosion mechanism is slow, but leads to well-infiltrated agglomerates and is not expected to cause damage to the tubes. Hansen et al. [31] proposed a criterion for determining whether the dispersion of agglomerates was through rupture or erosion. Briefly, if the dispersion stress is smaller than the cohesive strength of agglomerates, the agglomerate particles will be dispersed mainly through erosion. Otherwise, the agglomerates will be dispersed through the rupture mechanism.

The shear force applied to the mixture was calculated using Equation (1) as follows:

(1) $$\tau_{\mathrm{applied}}=K^{\prime }\eta \dot{\gamma },$$

where $K^{\prime }$ represents the shape factor, taken as 2.5 for spherical particles, and η is the viscosity of the mixed solution which was measured using a viscometer. The shear force obtained here was the maximum value because of the shear thinning effect of the polymer. The shear rate $\dot{\gamma }$ was calculated using Equation (2) as follows:

(2) $$\dot{\gamma }=\frac{V}{\delta }=\frac{\pi DN}{60\delta },$$

where D is the rotor diameter (19 mm), δ represents the clearance between the stator and rotor (0.5 mm), N is the mixing speed in rpm, and the rotational speed selected in the test was 10,000 rpm. Higher speeds in actual use can result in a faster temperature rise, which can exceed the cooling capacity of the tank. High temperatures are not conducive for good dispersion. The shear force of high-speed shear was calculated to be about 0.1 MPa.

The cohesion strength of agglomerates was calculated using the Rumpf equation [33], which indicates that the cohesion is mainly related to the size of the particles that make up the agglomerates. For spherical particles, the Rumpf equation (Equation (3)) is as follows:

(3) $$\sigma =\frac{\left(1-\varepsilon \right)}{\varepsilon }\frac{F}{a^{2}},$$

For irregular convex particles, the equation (Equation (4)) is expressed as follows:

(4) $$\sigma =\left(1-\varepsilon \right)k\frac{F}{A},$$

where σ is the cohesion strength, ε is the porosity of MWCNT agglomerates (0.85 [30]), k is coordination number (10 for rigid rods with an aspect ratio of 100 and packed with maximum density [34]), a is the diameter of particles forming the agglomerates, and A is the surface area. F represents the adhesive force which is predominantly van der Waals force between particles in the case of dry agglomerates, which can be calculated using Equations (5) and (6).

The van der Waals force between two spheres is given by

(5) $$F_{\mathrm{Sphere}}=\frac{-H}{6D^{2}}\left(\frac{R_{1}R_{2}}{R_{1}+R_{2}}\right),$$

while the van der Waals force between two rods/cylinders is given by

(6) $$F_{\mathrm{Cylinder}}=\frac{-H}{8\sqrt{2}}\frac{L}{D^{5/2}}{\left(\frac{R_{1}R_{2}}{R_{1}+R_{2}}\right)}^{1/2},$$

where H is Hamaker’s constant of MWCNT (60 × 10^−20^ J [35]), D is the inter-particle distance (assumed as 0.4 nm (inter graphite layer separation) [30]), and L and R represent the length and radius of the particles, respectively.

The aspect ratio, van der Waals force, and cohesion strength of the five kinds of C-MWCNTs are shown in Table 4. Based on the mechanisms of rupture and erosion, it can be reasonably inferred that the van der Waals force reflects the degree of difficulty of erosion to some extent, while the cohesion strength indicates that of rupture. The van der Waals forces are strongly influenced by the CNT surface chemistry, such as the presence of carboxylic groups, which generate negative surface charges on the MWCNTs [36,37]. Therefore, repulsive Coulombic force emerges between carbon nanotubes, and the value of the Coulombic force is a function of the distance between negatively charged nanotubes. The model of the interaction between nanotubes and nanoparticles, presented in a previous study [37], can be used to explain the interaction of carboxylic MWCNTs. The interactions are governed by both repulsive Coulombic and attractive van der Waals forces, and these two forces are balanced with the accumulation of electric charge. Because of the existence of repulsion force, the critical distance between carbon nanotubes becomes larger, whereby the structure of agglomerates is relatively loose and the compatibility with solvents can also be increased, which is helpful for the dispersion process. However, as a major hindrance to the mechanism of erosion dispersion, the van der Waals force is still regarded as the main criterion for the difficulty degree of erosion dispersion.

Therefore, according to the values in Table 4, the dispersion difficulty can be sorted as follows: (50, 8–15) > (10–30, 20–30) > (0.5–2, 20–30) ≈ (0.5–2, 50). The rank of C-MWCNTs with a length of 0.5–2 μm and a diameter of 8 nm is still unknown. However, it can be concluded that the C-MWCNT with a length of 50 μm and a diameter of 8–15 nm is the most difficult to disperse effectively, whether it is ruptured or eroded, and the short C-MWCNTs are easier to disperse through erosion. The sequencing is a theoretical speculation which is verified in next section. In addition, the result shows that the shear force produced by the dispersion equipment was much lower than the cohesion of the agglomerates, especially when the bundles were formed. Two assumptions were made by a previous report [20] for the dispersion process of CNT agglomerates. Firstly, after dispersing for a certain amount of time, the size of the agglomerates is reduced to a certain size, D_c_. The size of the agglomerates formed by highly dense carbon nanotubes (CNTs) with greater cohesion strength is not significantly reduced. Thus, the agglomerates are only affected by erosion mechanisms. Secondly, the larger agglomerates are formed by small agglomerates of the D_c_ class. The larger agglomerates rupture into smaller agglomerates under sufficient shear stress because the cohesion of the larger agglomerates is relatively weak. According to Figure 2 and the above analysis, the assumptions are reasonable. Therefore, taking the shear force as the cohesive strength of agglomerates, the theoretical minimum size of agglomerates dispersed through the rupture mechanism in this experiment can be deduced using Equations (3) and (5). The calculated result was 1–10 μm. For the further study of dispersion, the detailed macroscopic mechanical performance analysis is discussed below.

### 3.3. Tensile Properties

The tensile properties and related data of the eight different samples are shown in Table 5. Compared with neat epoxy, the addition of carbon nanotubes improved the tensile modulus of the resin to different extents, and the maximum increase was almost 20%. However, the strength did not change significantly, and the maximum increase was less than 8%. The increase in tensile modulus can be attributed to the formation of percolation networks by carbon nanotubes with large aspect ratios and specific surface areas. The modulus of the nanocomposites can be predicated using the well-established Halpin–Tsai model [38], which includes simple approximate forms of the generalized self-consistent micromechanics solutions developed by Hill [39]. The modulus values based on these equations agree reasonably well with the experimental values for a variety of reinforcement geometries, including fibers and flakes. The MWCNT nanocomposites were considered as randomly oriented discontinuous fibers, and were evaluated using Equation (7).

(7) $$E_{C}=\frac{3}{8}\left[\frac{1+\zeta \eta_{L}V_{\mathrm{NT}}}{1-\eta_{L}V_{\mathrm{NT}}}\right]E_{M}+\frac{5}{8}\left[\frac{1+2\eta_{T}V_{\mathrm{NT}}}{1-\eta_{T}V_{\mathrm{NT}}}\right]E_{M},\;\zeta =2\frac{l_{\mathrm{NT}}}{d_{\mathrm{NT}}},\;\eta_{L}=\frac{E_{\mathrm{NT}}/E_{M}-1}{E_{\mathrm{NT}}/E_{M}+\zeta },\;\eta_{T}=\frac{E_{\mathrm{NT}}/E_{M}-1}{E_{\mathrm{NT}}/E_{M}+2},$$

where $E_{\mathrm{NT}}$ is the equivalent modulus of nanotubes. The carbon nanotubes prepared using the CVD method contain a large number of defects. The research of Xie et al. suggests that the modulus is very sensitive to defects [40]; thus, the CVD-MWNTs are expected to display significantly reduced values. Here, $E_{\mathrm{NT}}$ = 40 GPa [27]. $E_{M}$ is the tensile modulus of the epoxy matrix and the value is shown in Table 5. $V_{\mathrm{NT}}$ is the volume content of the nanotubes which can be expressed as Equation (8) [41].

(8) $$V_{\mathrm{NT}}=\frac{w_{\mathrm{NT}}}{w_{\mathrm{NT}}+(\rho_{\mathrm{NT}}/\rho_{\mathrm{MER}})-(\rho_{\mathrm{NT}}/\rho_{\mathrm{MER}})w_{\mathrm{NT}}},$$

where $w_{\mathrm{NT}}$ is the mass fraction of carbon nanotubes, and $\rho_{\mathrm{NT}}$ and $\rho_{\mathrm{MER}}$ represent the densities of the carbon nanotubes and the matrix, respectively.

The calculated results are shown in Table 5, and the deviations between experimental and calculated values of tensile modulus are also listed in the table. It is obvious that the tensile modulus values of CNT-reinforced epoxy resin were predicted well by the model. Outside of samples No. 4 (10–30, 20–30, 1) and No. 8 (50, 8–15, 0.5), the deviations between predicted and experimental values were below 7%. The fairly high prediction accuracy indicates that the samples achieved good or acceptable dispersion. Among them, the deviations of samples No. 6 (0.5–2, 20–30, 0.5) and No. 7 (0.5–2, 50, 0.5) were 1.15% and 2.14%, respectively. This is due to their good dispersibility and is consistent with the previous evaluation of dispersion in Section 3.2. The deviation of sample No. 3 (10–30, 20–30, 0.5) was slightly higher (6.3%), while that of No. 8 (50, 8–15, 0.5) reached 18.35%. Comparing the samples with the same loading (0.5 wt. %), it was found that the magnitude of the deviation is related to the evaluation of dispersion in Section 3.2. Therefore, the deviation can be regarded as a criterion of dispersion. Similarly, we can deduce that carbon nanotubes with a length of 0.5–2 μm and a diameter of 8 nm have a moderate dispersion difficulty among the five kinds of carbon nanotubes, and the loading has great influence on the dispersion.

The large deviations of samples No. 4 and No. 8 are probably due to the excess loading of MWCNTs (10–30, 20–30, 1) and the aspect ratio (50, 8–15, 0.5). The dispersion of carbon nanotubes with a length of 50 μm and diameter of 8–15 nm was rather poor, as mentioned earlier. Thus, there would be a certain number of agglomerates in sample No. 8 (50, 8–15, 0.5). The resin entered into the voids of agglomerates to form sphere-like particles with large volume fractions of carbon nanotubes. The presence of agglomerates in the composites led to a decrease in the efficiency of stress transfer and the appearance and propagation of cracks in the interface between agglomerates and the matrix, resulting in a decrease in tensile modulus and strength. Compared with samples No. 2, No. 3, and No. 4, the tensile modulus and strength showed a trend of initial increase followed by decrease upon an increase in loading, which is consistent with a previous report [17]. The results of sample No. 4 (10–30, 20–30, 1) showed that there was also a problem of agglomerates when the loading was 1 wt. %. As the loading of carbon nanotubes was small, the dispersion of carbon nanotubes was better and the reinforcement effect was remarkable. With the increase in loading of carbon nanotubes, the number of agglomerates increased. 

**Figure 3:**
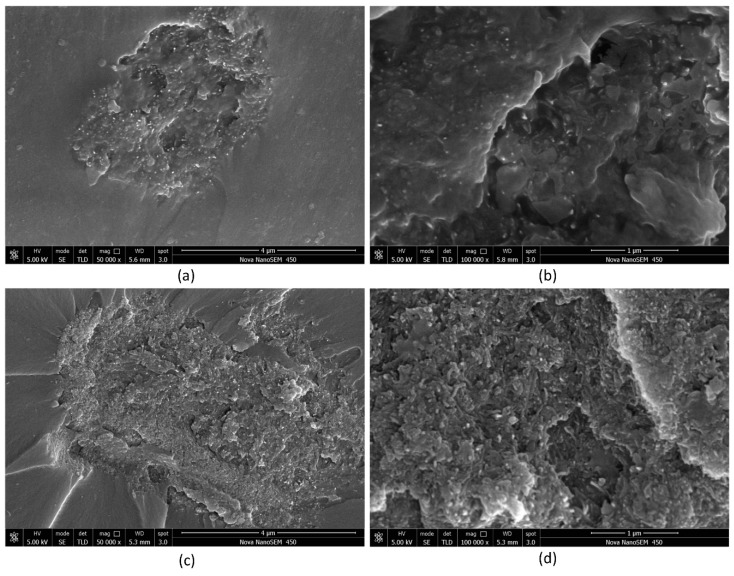
Agglomerates on the fracture surfaces of samples No. 8 (50, 8–15, 0.5) (**a**) and No. 4 (10–30, 20–30, 1) (**c**); internal morphology of the corresponding agglomerates (**b**,**d**).

**Figure 4:**
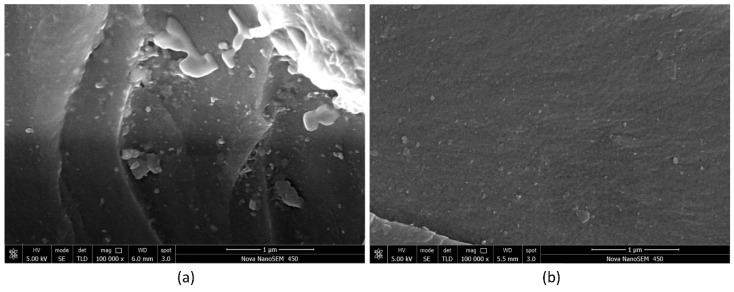
The fracture surfaces of samples of No. 3 (10–30, 20–30, 0.5) (**a**) and No. 5 (0.5–2, <8, 0.5) (**b**).

**Figure 5:**
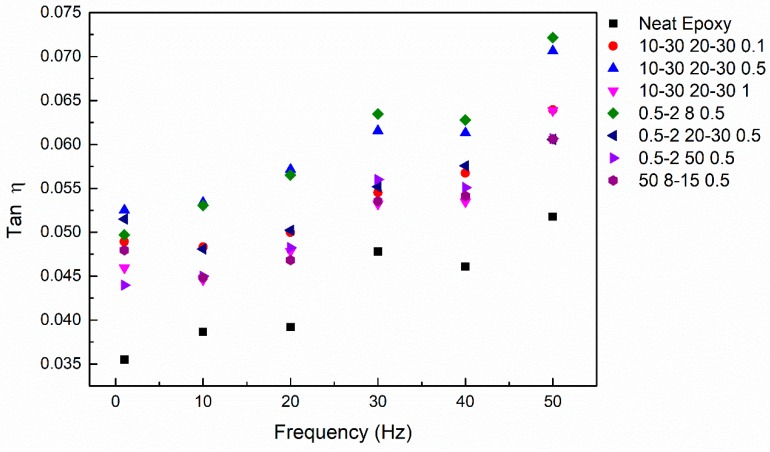
Loss factor of all samples at the frequency range of 1–50 Hz.

The fracture surfaces of the samples were investigated using SEM. As mentioned earlier, some agglomerates were observed on the fracture surfaces of samples No. 4 and No. 8, as shown in Figure 3. Compared with sample No. 8 (Figure 3a,b), the agglomeration of sample No. 4 (Figure 3c,d) was more severe. The calculated results of other samples (except for samples No. 4 and No. 8) were in good agreement with the experimental results. This result indicated that the carbon nanotubes were well dispersed, and the mechanical properties of the carbon nanotubes were fully utilized. When the cohesive strength is far greater than the dispersed shear force, it could be due to a dispersion effect (erosion) following magnetic stirring for a long time (8–12 h) after the high-speed shear dispersion. As shown in Figure 4, there were few agglomerates on the fracture surfaces of samples No. 3 (Figure 4a) and No. 5 (Figure 5b), and the distribution of MWCNTs was relatively uniform.

Comparing samples No. 3 and No. 6, it was found that the longer carbon nanotubes had higher modulus and strength. This is because longer carbon nanotubes facilitate the formation of permeation networks, and is also due to the stress transfer between carbon nanotubes and the matrix. For short-cut fibers, stress transfer between fibers and matrix must be considered. The maximum stress of the matrix to the carbon nanotubes is the interfacial shear strength. The amount of stress transferred varies with fiber length (*L*), such that, at some critical length (*L_c_*), the stress transferred is large enough to break the fiber. For a hollow cylinder, this critical length is given by Equation (9) [13].

(9) $$L_{c}=\frac{\sigma_{f}D}{2\tau }\left[1-\frac{D_{i}^{2}}{D^{2}}\right],$$

where $\sigma_{f}$ is the fiber strength, τ is the shear strength of the interface, and D and $D_{i}$ are the external and internal diameters of the fiber, respectively. Assuming an interfacial shear strength of 50 MPa and a strength of 10 GPa for CVD-MWCNTs, the critical length *L_c_* was calculated and the results are shown in Table 5. The length of most short carbon nanotubes (0.5–2 μm) was distributed below *L_c_*. As a result, the strength of carbon nanotubes cannot be fully exerted, while the enhancement effect of carbon nanotubes was not obvious, and it even slightly decreased. Comparing the data of samples No. 5, No. 6, and No. 7, it was found that the tensile modulus decreased with the increase in diameter, while the loading and length of CNTs were the same. In addition to the larger aspect ratio mentioned above, the large specific surface area of sample No. 5 (>500 m^2^/g) also contributed to this result. In other words, the enhancement effect of carbon nanotubes on the epoxy was influenced by both the dispersion and the technical parameters of carbon nanotubes. When the dispersion is limited by the technical parameters, the appropriate technical data are essential for the enhancement.

### 3.4. Damping Properties

As shown in Figure 5, the loss factors of C-MWCNT-reinforced nanocomposites increased to varying degrees at different frequencies compared with the neat epoxy. The stick-slip frictional motion of the interface between the nanotubes and the matrix is considered to be the main source of interface damping [11]. MWCNTs have many characteristics, such as large specific surface area, multilayer cavity structure, and nano-scale size, which can introduce a large number of interfaces into the epoxy resin. More interfaces also mean a greater loss factor. The critical shear stress is the key parameter affecting the stick-slip frictional motion [11]. In this study, weak interaction interfaces were formed between the carboxylic carbon nanotubes used and the epoxy matrix, which resulted in a smaller critical shear stress. Consequently, a larger deformation occurred at the interface under external force. In addition, the excellent damping properties of carbon nanotubes also help enhance the damping of nanocomposites [10]. The loss factor of each sample increased with frequency, in accordance with the established relationship between frequency and loss factor [42].

**Figure 6:**
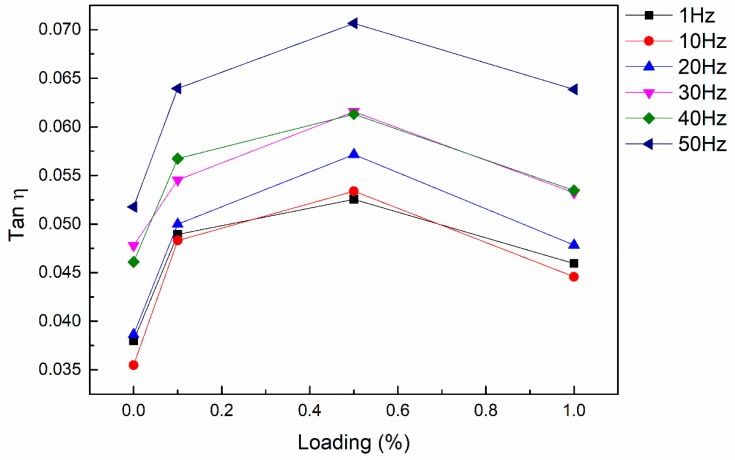
Relationship between loss factor and loading of MWCNTs at the frequency range of 1–50 Hz.

The loss factor of nanocomposites with different loading amounts is shown in Figure 6. With the increase in loading amount, the loss factor increased firstly and then decreased, which was also related to the dispersion of carbon nanotubes in the matrix. At lower filler percentages, there was a better distribution of MWCNTs within the matrix. With an increase in filler percentage, the MWCNTs dispersed more randomly in the resin and, therefore, the formation of local MWCNT clusters increased. The adhesion characteristics of MWCNTs and the epoxy matrix started deteriorating, resulting in interfacial slippage occurring under such circumstances. Alva et al. [43] reported that CNT agglomerations likely contribute to damping due to mutual slippage between the CNTs. However, this effect of agglomerates on damping was not consistent with the experimental results in this paper. The method of dispersion reported in the previous work [43] involved manual stirring for 15 min. This method can hardly open the agglomerates, even with the lowest addition amount, and it is also difficult to control the consistency of dispersion. It is reasonable to believe that the increase in loss factor with 0.75 wt. % loading in their experiment was probably due to the macroscopic defects caused by the excess agglomerates. The agglomerates in the poorly dispersed MWCNT nanocomposites can act as large spherical particles if higher filler loading amounts are present. Compared with uniformly dispersed carbon nanotubes, the interface between MWCNTs and the matrix is directly reduced. Moreover, the specific surface area of the spherical particles is relatively small, which further reduces the interface contact. Additionally, the spherical particles or near-spherical particles are less flexible compared to the well-dispersed carbon nanotubes, and still have a higher modulus than the surrounding matrix. Thus, the particles do not easily deform during vibrations, which leads to reduced deformation within the particles. This further reduces the internal interface slip of the particles, and ultimately causes a decrease in the loss factor. Therefore, agglomerates play a negative role in damping.

**Figure 7:**
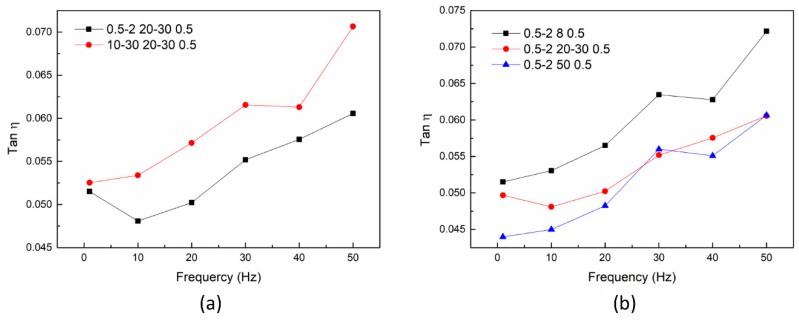
Relationship between loss factor and dimensions of MWCNTs at the frequency range of 1–50 Hz: length (**a**); diameter (**b**).

The relationship between the length of carbon nanotubes and the loss factor is shown in Figure 7a. The specific surface area, loading, and carboxyl functional group content of these two carbon nanotubes were identical. The longer carbon nanotubes showed higher loss factors. The results of Section 3.2 and Section 3.3 showed that the dispersion uniformity of these two carbon nanotubes was basically the same. Hence, nanocomposites with higher aspect ratios of C-MWCNTs were highly effective in enhancing the damping of the epoxy matrix. This is likely due to a similar reason as for the reduction of T_g_. The longer carbon nanotubes had stronger resistance for the cross-linking of epoxy resins and prevented entanglement between the molecular chain segments of the matrix, which provided a larger free volume for the movement of the molecular chains of the matrix and facilitated the movement of molecular chain segments of the matrix with vibration. The relationship between the diameter of carbon nanotubes and the loss factor is shown in Figure 7b. The smallest-diameter CNT-reinforced nanocomposite (0.5–2, 8, 0.5) exhibited the largest loss factor, and this result corresponds well with the mechanism of damping enhancement of CNT-reinforced nanocomposites. In addition to the larger aspect ratio, the smallest-diameter carbon nanotubes had the largest specific surface area and content of carboxyl groups. In Section 3.4, both samples No. 3 (10–30, 20–30, 0.5) and No. 5 (0.5–2, 8, 0.5) with the greatest decrease in T_g_ displayed the largest loss factor, which is in good agreement with the above analysis. 

**Figure 8:**
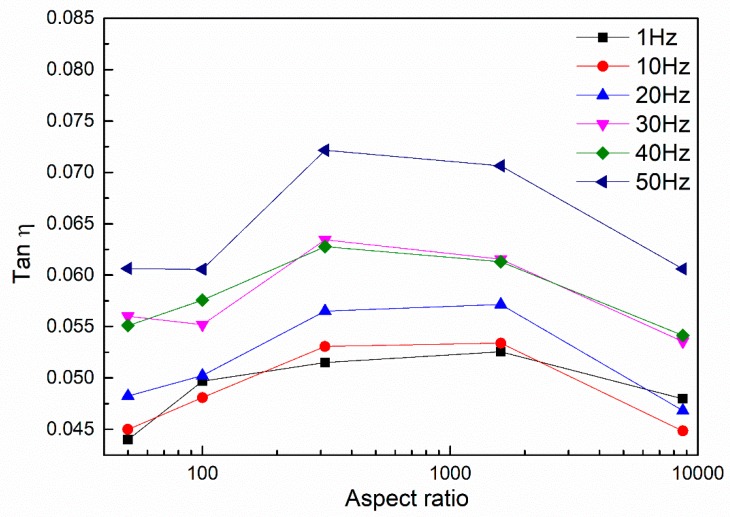
Relationship between loss factor and aspect ratio of MWCNTs at the frequency range of 1–50 Hz.

Taking the loss factor of different samples as the vertical coordinate and the logarithm of aspect ratio as the horizontal coordinate, the relationship between the aspect ratio of C-MWCNTs and loss factor is shown in Figure 8. With the increase in aspect ratio, the loss factor of C-MWCNT-reinforced nanocomposites firstly increased and then decreased. The MWCNTs of sample No.8 (50, 8–15, 0.5) had the largest aspect ratio, but the loss factor was equal to that of No. 7 (0.5–2, >50, 0.5) which confirmed the negative effect of carbon nanotube agglomerates on loss factors. Compared with the loss factors of samples No. 3 (0.5–2, 8, 0.5) and No. 5 (10–30, 20–30, 0.5), the loss factor of No. 5 was slightly higher than that of No. 3 at low frequency. However, with the increase in frequency, the loss factor of sample No. 3 exceeded that of No. 5, which was probably due to its higher specific surface area. As the frequency increased, more interface slippages were activated, thus increasing the loss factor.

### 3.5. T_g_ of Nanocomposites

As shown in Table 6, the glass transition temperature (T_g_) value of the nanocomposites decreased with the addition of C-MWCNTs in varying degrees compared with the neat epoxy. The T_g_ of polymers is closely related to the movement of polymer molecular chain segments. There are two reasons for the decrease in glass transition temperature. Firstly, the steric limitation and the phase segregation of C-MWCNTs are likely to cause the functional groups of matrices near the surface to be unable to react, because the presence of the nanotube limits the number of functional groups in the local vicinity, and the local functional group ratios are not consistent with their values of the bulk matrix. Therefore, it leads to the disruption of the matrix network near the surface and the effective reduction of cross-linking within the matrix [44]. The decrease in the entanglement of chains near the C-MWCNT surface allows the polymer chain to move easily, resulting in the decrease in T_g_. Secondly, there is no reaction between the carboxylic functional group and the epoxy resin, and the interface bonding is also mainly dependent on van der Waals forces. The interphase with weak interfacial bonding leads to the presence of a very small layer of matrix atoms between non-interacting nanotubes, which act as a polymer thin film. The interface confines the thin-film region, and the weak interfacial bonding provides a larger free volume for the thin film (epoxy matrix), which causes the decrease in T_g_ [45]. Both of these effects reduced the T_g_. In addition, the amine curing agent used in the curing process could react with the carboxyl group. However, considering the small addition of carbon nanotubes (1 wt. % at most), the content of functional groups should be lower (0.49–3.86 wt. %). Thus, there was little effect on T_g_.

**Figure 9:**
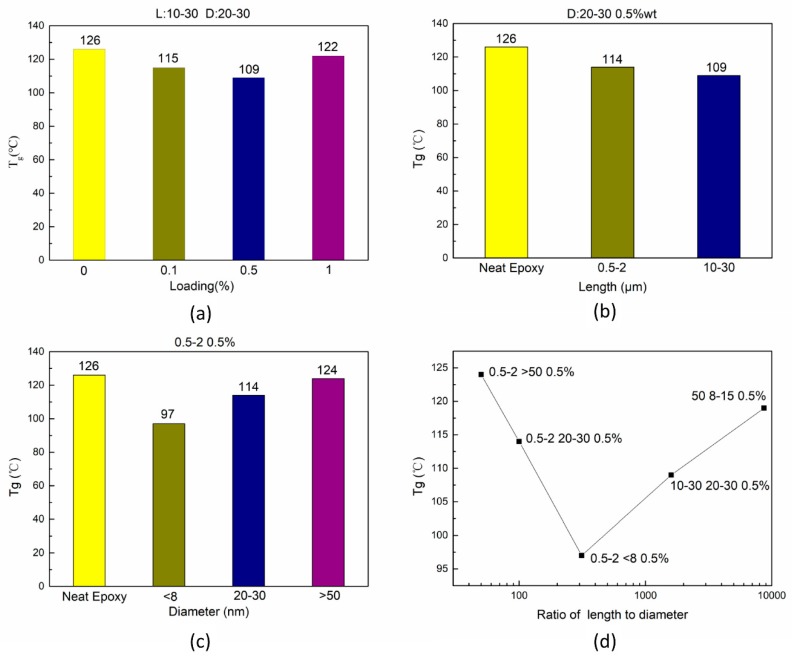
Th relationship between T_g_ and loading (**a**); length of MWCNTs (**b**); and diameter of MWCNTs (**c**); the ratio of length to diameter (**d**).

The relationship between loading of carbon nanotubes and T_g_ is shown in Figure 9a. With the increase in loading, T_g_ decreased firstly and then increased. The loading of carbon nanotubes mainly affected the T_g_ of nanocomposites based on the state of dispersion of carbon nanotubes and the content of carboxyl groups in the unit volume. When the mass fraction of C-MWCNT was 1 wt. %, the C-MWCNTs in the unit volume should be higher than 0.1 wt. % and 0.5 wt. %. However, the increase in loading led to the deterioration of dispersion. Therefore, the number of carbon nanotubes in the form of agglomerates increased, which greatly reduced the efficiency of C-MWCNTs in forming weak interaction interfaces. Compared with neat epoxy, the T_g_ of sample No. 4 (10–30, 20–30, 1) decreased by only 4 °C and was higher than that of sample No. 2 (10–30, 20–30, 0.1) and sample No. 3 (10–30, 20–30, 0.5).

The relationship between the length of carbon nanotubes and T_g_ is shown in Figure 9b. When the loading, diameter, specific surface area, and the content of carboxyl groups were the same, the T_g_ of the sample containing longer carbon nanotubes (10−30, 20–30, 0.5) was lower than that of the sample with short carbon nanotubes. During the curing process, the presence of C-MWCNTs with large aspect ratio played a stronger role in hindering the cross-linking of epoxy molecular segments near the surface, better prevented the entanglement of molecular chain segments, provided a larger free volume for the movement of the epoxy matrix segment, and enabled the formation of a larger area with lower cross-linking degree, which ultimately led to lower values of T_g_. The relationship between the diameter of carbon nanotubes and T_g_ is shown in Figure 9c. The T_g_ of sample No. 5 (0.5–2, 8, 0.5) decreased dramatically, and the T_g_ of sample No. 7 (0.5–2, 50, 0.5) decreased by only 2 °C compared to neat epoxy. Under the same loading, dispersion, and length distribution, the MWCNTs with smaller diameter showed larger aspect ratios and specific surface areas, and contained more carboxyl groups per unit volume. As mentioned above, large aspect ratios and more carboxyl groups exert a negative effect on T_g_. The larger specific surface area enabled carbon nanotubes to form more weak interaction interfaces with the matrix, which also played a negative role. Thus, the T_g_ of sample No. 5 was the smallest among all the samples.

Taking the T_g_ of different samples as the vertical coordinate and the logarithm of aspect ratio as the horizontal coordinate, the relationship between the aspect ratio of C-MWCNTs and T_g_ is shown in Figure 9d. With the increase in aspect ratio, T_g_ decreased initially and then increased. This trend was not only determined by aspect ratio, but also related to specific surface area, carboxyl content, and dispersion. In particular, the ratio of length to diameter, specific surface area, and carboxyl group content of carbon nanotubes for sample No. 8 (50, 8–15) were 8695, 233 m^2^/g, and 2.56 wt. %, respectively, which were larger than those of other carbon nanotubes. Nevertheless, the T_g_ of sample No. 8 was only slightly lower than that of neat epoxy, which was obviously due to poor dispersion.

## 4. Conclusions

In this paper, the effects of dimensions and agglomerations of MWCNTs on the synchronous enhancement of mechanical properties and damping properties were studied. A reasonable dispersion process to ensure the uniformity and stability of dispersion was selected, and then the study on the dispersion mechanism was carried out using theoretical analysis. The difficulty of dispersion of MWCNTs with different dimensions was evaluated. The effect of synchronous enhancement was tested experimentally. Furthermore, the effects of carbon nanotube loading, aspect ratio, specific surface area, and functional groups on the properties of nanocomposites were analyzed.

The morphologies of agglomerates of MWCNTs prepared by CVD with different lengths were considerably different. The van der Waals force and cohesion strength were attributed to the degree of difficulty of dispersion via the mechanisms of erosion and rupture. On the whole, the dispersion of carbon nanotubes with a diameter of 8–15 nm and a length of 50 μm was the worst. The minimum size of agglomerates obtained through the mechanism of rupture was determined to be 1–10 μm by back-calculation. The ranking of dispersion difficulty was verified by the deviations between predicted and experimental values of tensile modulus. Among all the samples, No. 3 (10–30, 20–30, 0.5) and No. 5 (0.5–2, 8, 0.5) had the best effects of synchronous enhancement, but their T_g_ showed the maximum decrease. It was found that a large aspect ratio and high specific surface area improved the mechanical properties and damping properties synchronously, but decreased the T_g_ of nanocomposites. The agglomerations had a negative effect on the synchronous enhancement of the nanocomposites, but reduced the negative impact on T_g_. Overall, C-MWCNTs with appropriate technical parameters can simultaneously improve the mechanical properties and damping properties of epoxy resin under appropriate dispersion conditions, but the T_g_ will decrease to a certain extent.

## Figures and Tables

**Table 1:** Technical parameters of carbon nanotubes.

	Length (μm)	OD ^1^ (nm)	ID ^2^ (nm)	SSA ^3^ (m^2^/g)	Purity (%)	Functional Group	Group Content (wt. %)
1	10–30	20–30	5–10	>110	>95	–COOH	1.23
2	0.5–2	<8	2–5	>500			3.86
3	0.5–2	20–30	5–10	>110			1.23
4	0.5–2	>50	5–15	>40			0.49
5	50	8–15	3–5	>233			2.56

^1^ OD = outer diameter; ^2^ ID = inner diameter; ^3^ SSA = specific surface area.

**Table 2:** Properties of epoxy resin (HT723).

Property	Value
Viscosity at 25 °C (mPa·s)	1100 ± 200
Curing agent	Amines
Viscosity of mixed at 25 °C (mPa·s)	250 ± 50
Operable time (min)	80
T_g_ (°C)	115–125
Tensile strength (MPa)	74.5
Tensile modulus (GPa)	2.65
Flexural strength (MPa)	117.67
Flexural modulus (GPa)	3.2
Compressive strength (MPa)	105

**Table 3:** Type and loading of carbon nanotubes (CNTs) of all samples. The code is in the format of “length, diameter, loading”.

	Code	Length (μm)	OD (nm)	CNT Loading (wt. %)
1	Neat Epoxy	-	-	-
2	10–30, 20–30, 0.1	10–30	20–30	0.1
3	10–30, 20–30, 0.5	10–30	20–30	0.5
4	10–30, 20–30, 1	10–30	20–30	1
5	0.5–2, 8, 0.5	0.5–2	<8	0.5
6	0.5–2, 20–30, 0.5	0.5–2	20–30	0.5
7	0.5–2, 50, 0.5	0.5–2	>50	0.5
8	50, 8–15, 0.5	50	8–15	0.5

**Table 4:** Aspect ratios, van der Waals forces, and cohesive force of carbon nanotubes.

CNT Type	10–30, 20–30	0.5–2, 8	0.5–2, 20–30	0.5–2, 50	50, 8–15
Aspect ratio	1600	312	100	50	8695
F (N)	−2.6204 × 10^−5^	−9.2645 × 10^−7^	−1.6378 × 10^−6^	−2.3161 × 10^−6^	−4.4431 × 10^−5^
σ (MPa)	−25.0229	−44.2347	−25.0229	−17.6939	−36.8943

**Table 5:** Tensile Properties of multi-walled CNT (MWCNT)-reinforced nanocomposites.

	Materials	Tensile Modulus/MPa			Tensile Strength (MPa)	*Lc* (μm)
		Experiment	Calculation	Deviation (%)		
1	Neat epoxy	2666.28			74.36	
2	10–30, 20–30, 0.1	2753.72	2833.56	2.90.	75.46	1500–2916.67
3	10–30, 20–30, 0.5	3177.84	3378.17	6.30	78.46	1500–2916.67
4	10–30, 20–30, 1	2890.66	4157.26	39.59	72.13	1500–2916.67
5	0.5–2, <8, 0.5	3030.09	3162.90	4.38	72.30	487.5–750
6	0.5–2, 20–30, 0.5	2931.09	2964.60	1.15	72.07	1500–2916.67
7	0.5–2, >50, 0.5	2753.90	2812.87	2.14	75.25	4550–4950
8	50, 8–15, 0.5	2959.17	3443.02	18.35	72.66	487.5–1440

**Table 6:** Glass transition temperature of all samples.

	Code	T_g_ (°C)
1	Neat Epoxy	126
2	10–30, 20–30, 0.1	115
3	10–30, 20–30, 0.5	109
4	10–30 20–30, 1	122
5	0.5–2, 8, 0.5	97
6	0.5–2, 20–30, 0.5	114
7	0.5–2, 50, 0.5	124
8	50, 8–15, 0.5	120
